# Functional association of *NR4A3* downregulation with impaired differentiation in myeloid leukemogenesis

**DOI:** 10.1007/s00277-022-04961-1

**Published:** 2022-08-30

**Authors:** Shih-Chiang Lin, Chi-Yuan Yao, Cheng-An Hsu, Chien-Ting Lin, Marcus J. Calkins, Yuan-Yeh Kuo, Jih-Luh Tang, Hwei-Fang Tien, Shang-Ju Wu

**Affiliations:** 1 Department of Internal Medicine, Far-Eastern Memorial Hospital, New Taipei City, Taiwan; 2 General Education Center, Lunghwa University of Science and Technology, Taoyuan City, Taiwan; 3 Department of Laboratory Medicine, National Taiwan University Hospital, Taipei City, Taiwan; 4 Department of Internal Medicine, Zhongzheng Dist, National Taiwan University Hospital, No.7, Chung Shan S. Rd, Taipei City, 100225 Taiwan; 5 Tai-Cheng Cell Therapy Center, National Taiwan University Cancer Center, Taipei City, Taiwan; 6 Pell Bio-Med Technology CO., LTD., Taipei City, Taiwan; 7 Department of Hematological Oncology, National Taiwan University Cancer Center, Taipei City, Taiwan; 8 Institute of Cellular and Organismic Biology, Academia Sinica, Taipei City, Taiwan

**Keywords:** Orphan receptor, NR4A3, Differentiation, Acute myeloid leukemia

## Abstract

The coincident downregulation of *NR4A1* and *NR4A3* has been implicated in myeloid leukemogenesis, but it remains unknown how these two genes function in myeloid cells and how their combined downregulation promotes myeloid leukemogenesis. Since *NR4A1* abrogation is thought to confer a survival and proliferation advantage to myeloid cells, we hypothesized that downregulation of *NR4A3* may have a complementary effect on myeloid cell differentiation. First, we tested the association between differentiation status of leukemic cells and *NR4A3* expression using two large clinical datasets from patients with different acute myeloid leukemia (AML) subtypes. The analysis revealed a close association between differentiation status and different subtypes of AML Then, we probed the effects of differentiation-inducing treatments on *NR4A3* expression and *NR4A3* knockdown on cell differentiation using two myeloid leukemia cell lines. Differentiation-inducing treatments caused upregulation of *NR4A3*, while *NR4A3* knockdown prevented differentiation in both cell lines. The cell culture findings were validated using samples from chronic myeloid leukemia (CML) patients at chronic, accelerated and blastic phases, and in acute promyelocytic leukemia (APL) patients before and after all trans-retinoic acid (ATRA)-based differentiation therapy. Progressive *NR4A3* downregulation was coincident with impairments in differentiation in patients during progression to blastic phase of CML, and *NR4A3* expression was increased in APL patients treated with ATRA-based differentiating therapy. Together, our findings demonstrate a tight association between impaired differentiation status and *NR4A3* downregulation in myeloid leukemias, providing a plausible mechanistic explanation of how myeloid leukemogenesis might occur upon concurrent downregulation of *NR4A1* and *NR4A3.*

**Supplementary information:**

The online version contains supplementary material available at 10.1007/s00277-022-04961-1.

## Background

Myeloid leukemogenesis is driven by specific genetic mutations that uniquely contribute to oncogenic cellular phenotypes in the myeloid lineage [1]. For many years, researchers have known of several key recurrent genetic aberrations (i.e., certain translocations or gene disruptions) that not only play important roles in the development of acute myeloid leukemia (AML) but also impact treatment responses and disease prognosis. Based on the functional categorization of these well-known mutations, it was proposed that genetic changes driving AML can be classified into two major groups: *class I* aberrations that confer survival or proliferation advantages to myeloid blood cells and *class II* aberrations that block the normal differentiation process of blood cells [2–4]. Furthermore, the *two-hit theory* stipulates that a combination of at least one class I aberration and one class II aberration is necessary for development of the AML phenotype [5, 6]. In recent years, next-generation sequencing has facilitated the discovery of many other genetic anomalies in AML. While most of the newly identified genetic aberrations do not fit neatly into either of the two mutation classes, the combined genetic aberrations are thought to function via mechanisms that recapitulate leukemogenic effects on survival/proliferation and differentiation [7]. Thus, the two-hit model is still considered to be a relevant paradigm of myeloid leukemogenesis, and it remains a useful framework for investigating how AML can result from orchestrated genetic aberrations [1, 8].

Previously, Mullican et al. reported that two orphan receptor genes, *NR4A1* and *NR4A3*, possess unexpected suppression functions in myeloid leukemogenesis. As such, concurrent abrogation of both nuclear receptors in mice results in the development of an AML-like phenotype [9]. Even more importantly, the authors also showed that downregulation of each gene is a common feature in leukemic blasts from AML patients, irrespective of karyotype [9]. While these findings suggest that the silencing of both genes plays a key role in myeloid leukemogenesis [10], the impacts of individually downregulating *NR4A1* and *NR4A3* remain unclear. A few studies have linked *NR4A1* to apoptosis [11–14], but the functional effects of *NR4A3* downregulation in myeloid cells and neoplasms are largely unknown. In the process of T-cell apoptosis, *NR4A3* was shown to be functionally redundant with *NR4A1* [15, 16]. However, the genes may have non-overlapping functions in myeloid leukemogenesis. In support of this idea, the same group of researchers as those who produced the Mullican et al. study showed that knocking out asingle allele of either *NR4A1* or *NR4A13* gene alongside homozygous knockout of the other gene produced phenotypes consistent with mixed myelodysplastic/myeloproliferative neoplasms, revealing a gene-dose effect [17]. Notably, the neoplastic phenotypes differed depending on which gene was fully knocked out, suggesting that the two genes are likely to act as cooperative rather than redundant suppressors of myeloid leukemogenesis [17, 18].

Because the concurrent knock-out of *NR4A1* and *NR4A3* is sufficient to produce an AML-like phenotype in mice [9], we speculated that the loss of both genes should satisfy the dual requirements of the two-hit theory of myeloid leukemogenesis. *NR4A1* is thought to function in cell apoptosis, so its downregulation would be expected to confer a survival advantage to myeloid cells (i.e., class I aberration). Thus, *NR4A3* downregulation might be expected to function in a complementary manner, as a class II aberration that impairs cell differentiation. Indeed, our clinical and experimental data in this study support this idea, leading us to conclude that *NR4A3* downregulation likely contributes to myeloid leukemogenesis by interfering with myeloid differentiation.

## Methods

### Patient cohorts and primary patient samples

The National Taiwan University Hospital (NTUH) cohort included 227 AML patients. The transcriptomes of bone marrow cells from these patients were profiled using the Illumina HumanHT-12 v4.0 Expression BeadChip platform (Illumina, San Diego, CA, USA), as described previously [19] (Gene Expression Omnibus accession numbers GSE68469 and GSE71014). The expression data were log2 transformed and quantile normalized. The collection and analyses of patient samples were approved by the institutional review board of NTUH.

For the The Cancer Genome Atlas (TCGA) cohort, clinical and RNA-seq data from the TCGA-LAML cohort (*N* = 151) were downloaded from the TCGA website (https://portal.gdc.cancer.gov/). The gene expression data were normalized to Fragments Per Kilobase of transcript per Million mapped reads upper quartile (FPKM-UQ), and then a log2 transformation was applied.

The collection and use of archived marrow samples from patients with chronic myeloid leukemia (CML) and fresh blood samples from patients with acute promyelocytic leukemia (APL) were approved by the institutional review board of NTUH.

### Cell lines

The K562 cell line was obtained from the Bioresource Collection and Research Center (BCRC) and maintained in IMDM (GIBCO, Life Technologies Corporation, NY, USA) supplemented with 10% heat-inactivated fetal bovine serum (GIBCO, Life Technologies Corporation, NY, USA) and antibiotics (penicillin 100 U/ml, streptomycin 50 μg/ml; GIBCO, Life Technologies Corporation, NY, USA). NB4 cells were kindly provided by Prof. Wen-Chien Chou at National Taiwan University Hospital and were maintained in RPMI 1640 (GIBCO, Life Technologies Corporation, NY, USA) supplemented with 15% heat-inactivated FBS and antibiotics (penicillin 100 U/ml, streptomycin 50 μg/ml; GIBCO, Life Technologies Corporation, NY, USA). Both cell lines were grown in a 5% CO_2_ humidified atmosphere at 37 °C.

### Cell differentiation

K562 and NB4 cell lines were treated with various differentiation-inducing agents. Megakaryocytic differentiation of K562 cells was assayed by real-time quantitative PCR to measure expression of the platelet-derived growth factor A (*PDGFA*) gene and by morphological examination of the cells after staining with Liu’s stain. Myelo-monocytic differentiation of NB4 cells was evaluated by morphological examination after Liu’s stain and flow cytometry (BD FACSverse™) for CD11b expression with PE-conjugated CD11b antibody (BD Biosciences Pharmingen, San Diego, CA, USA) or IgG1 isotype control antibody (eBioscience, Thermo Fisher Scientific Inc.). Flow cytometry data were analyzed with BD FACSuite software or FlowJo™ software for Windows, version 10 (Becton Dickinson, and Company, Ashland). To exclude dead cells, 7-AAD (BD Biosciences Phamingen) was added to facilitate the gating procedure.

### RNA extraction and reverse transcription

Total RNA was extracted from the harvested cells using the NucleoSpin RNA mini Kit (Macherey–Nagel, GmbH & Co. KG, Germany). Residual genomic DNA was eliminated by treatment with RNase-free rDNase. Complementary DNA (cDNA) was generated by reverse transcription (RT) using the PrimeScript™ RT-PCR Kit (TAKARA Bio Inc., Japan).

### Real-time quantitative polymerase chain reaction

A total of 100 ng of cDNA was added to a real-time quantitative polymerase chain reaction (RT-qPCR) mixture containing 2 × TaqMan PCR Master Mix (Roche Life Science, USA), primers, and fluorescent probes or SYBR Green (Applied Biosystems, Foster City, CA, USA). PCRs were performed using the Applied Biosystems 7900HT Real-Time PCR System and Applied Biosystems Sequence Detection Systems (SDS v2.2.2) software (Applied Biosystems, Foster City, CA, USA). The primer sequences for *NR4A1* were forward: 5′-ACTTTGGGAAGGAAGATGCTG-3′, and reverse: 5′-TTCGGATGACCTCCAGAGAA-3′. To probe *NR4A3*, *PDGFA*, and *ABL1* (housekeeping gene) expression, Taqman Gene Expression Assays were purchased (Hs00545007, Hs00964426, and Hs01104728, respectively; Applied Biosystems, Foster City, CA, USA). All gene expression experiments in patient samples were repeated twice.

### Knockdown of NR4A3 expression

The SMARTpool ON-TARGETplus siRNA Set (catalog # LQ-003428–00) and the scrambled siRNA control used in K562 cells were purchased from Thermo Fisher Scientific (Lafayette, CO). The transfection was performed using the Neon™ Transfection System (catalog # MPK5000, Thermo Fisher Scientific., Lafayette, CO.) at settings of 1000 V, 50 ms, and 1 pulse. The sh-Luciferase control and two different shNr4A3 (D2-2 and E1-1) vectors used in NB4 cells were purchased from the RNAi Core of Academia Sinica, Taiwan. The shRNA target sequences were D2-2: GCAGACATACAGCTCGGAATA and E1-1: AGAAGATCAGACATTACTTAT. 293FT cells (Invitrogen, Life Technologies Corporation, NY, USA) were plated on 60-mm dishes and transfected with shNr4A3 or scrambled vector using Lipofectamine 2000 (Invitrogen, Life Technologies Corporation, NY, USA) to generate lentivirus. After transfection for 16–24 h, the medium was replaced with fresh complete medium containing 1% bovine serum albumin (BSA). Then, the supernatant was collected 24 and 48 h later. The supernatant, which contained lentivirus particles, was concentrated and cryopreserved at − 80 ℃. K562 or NB4 was seeded on 6-well plates, infected with concentrated lentivirus, and treated with puromycin for selection (Sigma-Aldrich). Total cell lysates were collected from K562 or NB4 cells to assess *Nr4A3* gene expression. All data were derived from at least three independent experiments.

### Statistical analysis

Data are represented as mean ± standard error of the mean (sem) unless otherwise specified. For measurements from clinical samples, statistical significance of differences between groups was determined by the Kruskal–Wallis test. For comparisons to the control group in cell culture experiments, paired Student’s *t*-test was used (two-tailed). *p*-values less than 0.05 were considered significant for all analyses.

## Results

### NR4A3 expression is associated with differentiation status of leukemic cells in primary AML

To gain a basic understanding of the *NR4A3* expression profiles in normal myeloid cells, we examined the publicly available GSE42519 dataset (visualized at BloodSpot, http://www.bloodspot.eu/), which includes gene expression profiles for the normal myeloid lineage cells of the hematopoietic system. In these cell types, there was an obvious trend toward lower *NR4A3* expression levels in more immature cell populations (e.g., hematopoietic stem cells and multi-potent progenitors) compared to more differentiated populations (e.g., band cells, polymorphonuclear neutrophils, and monocytes) (Supplementary Fig. 1).

**Fig. 1:**
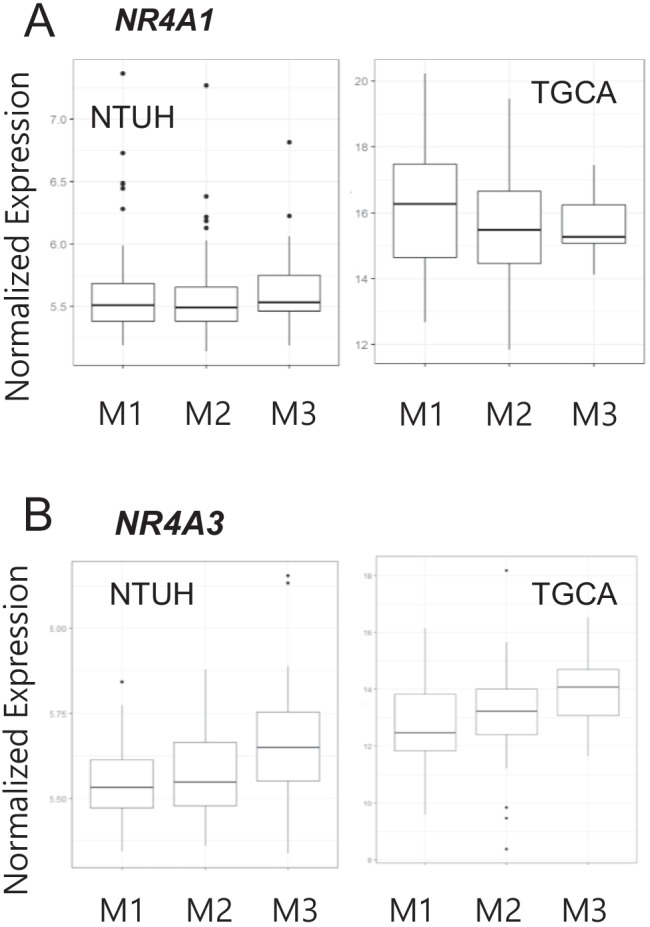
Normalized expression levels of *NR4A3* and *NR4A1* among M1, M2, and M3 AML subtypes. The expression levels of *NR4A3* are higher in subtypes with more differentiated status. **A** This pattern was observed in both the National Taiwan University Hospital cohort (*p* = 0.030) and **B** the TCGA dataset (*p* = 0.038). In contrast, the expression levels of *NR4A1* are similar among all three subtypes in both cohorts; **C** National Taiwan University Hospital (*p* = 0.400) and **D** TCGA dataset (*p* = 0.436)

We then analyzed the expression of *NR4A3* in 227 AML patients of the NTUH cohort [19]. Among these patients, the normalized *NR4A3* expression levels showed a graded changes according to the AML subtype. The expression levels were lowest in the M1 subtype (AML without maturation, morphologically), intermediate in the M2 subtype (AML with maturation, morphologically), and highest in the M3 subtype (acute promyelocytic leukemia; APL) (*p* = 0.030, Kruskal–Wallis H-test; Fig. 1A). Notably, a similar trend was found when analyzing *NR4A3* expression levels in the TCGA AML cohort. Again, the expression levels were gradually elevated from the least to most differentiated (M1 to M3) subtypes (*p* = 0.038, Kruskal–Wallis H-test; Fig. 1B). In contrast, the expression levels of *NR4A1* in the NTUH and TCGA cohorts did not show any discernable trends (Fig. 1C and D). These gene expression data from large cohorts supported the conclusion that *NR4A3* expression levels are inversely correlated with cell differentiation in different AML subtypes, while *NR4A1* expression is not.

### NR4A3 expression is upregulated by induction of cell differentiation

**Fig. 2:**
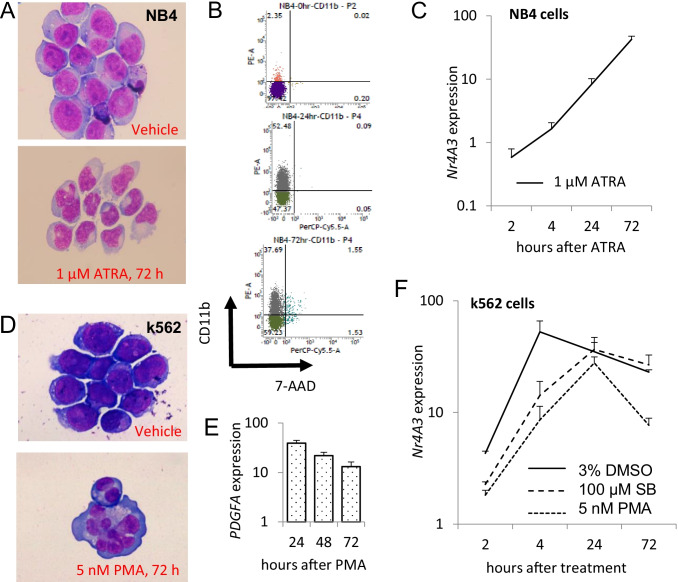
Upregulation of *NR4A3* expression upon induction of cell differentiation. **A** NB4 cells exhibited myelo-monocyte-like morphological features after 72 h treatment with ATRA. **B** The ATRA-treated NB4 cells also exhibited higher CD11b levels after 24 and 72 h, as measured by flow cytometry. CD11b and 7-AAD antibodies were respectively labeled with PE and PerCP-Cy5.5. **C** *NR4A3* transcripts (normalized to the pre-treatment level) were time-dependently elevated in NB4 cells after ATRA treatment. **D** After 72 h treatment with PMA, K562 cells exhibited (pro)megakaryocyte-like morphologies. **E** PMA-treated K562 cells had upregulated platelet-derived growth factor-A (PDGFA) as soon as 24 h, and for at least 72 h after treatment. **F** *NR4A3* transcripts (normalized to the pre-treatment level) were elevated in K562 cells after treatment with various differentiation induction agents. Abbreviations: ATRA, all-trans-retinoic acid; DMSO, dimethyl sulfoxide; SB, sodium butyrate; PMA, phorbol 12-myristate 13-acetate

We next took advantage of established protocols for chemical induction of differentiation in NB4 and K562 myeloid leukemia-derived cell lines [20–24] to test whether the *NR4A3* expression level would be upregulated upon differentiation. First, we treated NB4 cells with all-trans-retinoic acid (ATRA), which induces myeloid and monocytic differentiation [25]. Cellular differentiation and relative *NR4A3* expression levels were then measured at various time-points after treatment. ATRA-treated NB4 cells exhibited clear characteristics of myelo-monocytic differentiation, according to their morphology (Fig. 2A) and increased expression of CD11b (Fig. 2B). Moreover, *NR4A3* expression was also upregulated until at least 72 h post-treatment (Fig. 2C). We then sought to test whether similar results would be found for K562 cells, which can be differentiated into different lineages using different compounds. Differentiation of these cells may be induced by dimethyl sulfoxide (DMSO, myeloid, and monocytic differentiation), sodium butyrate (SB, erythroid differentiation), or phorbol 12-myristate 13-acetate (PMA, megakaryocytic differentiation) [26]. Notably, PMA-treated K562 cells exhibited morphological characteristics mimicking megakaryocytes (Fig. 2D), and compared to pre-treatment levels, the differentiated cells had *PDGFA* expression levels that were upregulated by at least tenfold up to 72 h post-treatment (Fig. 2E). Additionally, all differentiation-inducing treatments caused upregulation of *NR4A3* expression by 72 h post-treatment (Fig. 2F). These findings demonstrate that treatment of at least two different myeloid leukemia cell lines with various differentiation-inducing agents consistently upregulates *NR4A3* expression.

### Knockdown of NR4A3 impairs cell differentiation

**Fig. 3:**
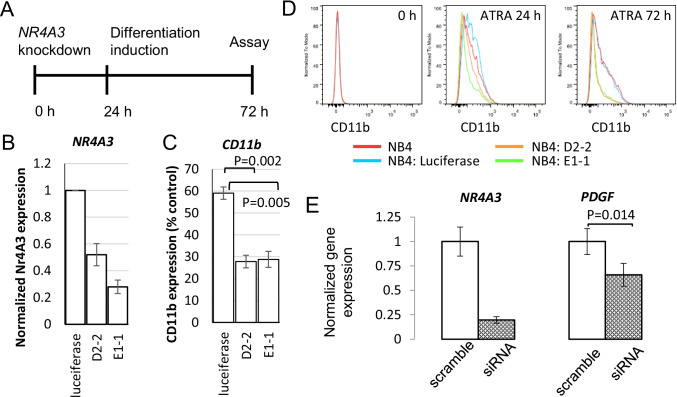
Knockdown of *NR4A3* expression impairs cell differentiation in NB4 and K562 cells. **A** Twenty-four hours after *NR4A3* knockdown with siRNA or shRNA, NB4 or K562 cells were treated with differentiation-inducing agents for another 72 h. Then, the cells were assayed for the differentiation phenotypes. **B** In NB4 cells, knockdown of *NR4A3* with shRNA suppressed post-ATRA treatment CD11b expression levels. D2-2: *p* = 0.002 and E1-1: *p* = 0.005, compared with the control group. **C** Flow cytometric measurements of CD11b after treatment with ATRA in NB4 cells with *NR4A3* knockdown. **D** In K562 cells, knockdown of *NR4A3* with siRNA significantly suppressed post-PMA treatment PDGFA expression; *p* = 0.014 compared with the control group

To further delineate the relationship between myeloid differentiation and *NR4A3* expression, we knocked down *NR4A3* with siRNA or shRNA prior to the induction of differentiation (Fig. 3A). After transfection of NB4 cells with *NR4A3* shRNA, the ATRA-induced *NR4A3* upregulation was abrogated (Fig. 3B). Concurrently, CD11b expression was suppressed (Fig. 3Cand D), suggesting that differentiation induction by ATRA was ineffective in cells with *NR4A3* knockdown (shNr4A3 D2-2, *p* = 0.002; shNr4A3 E1-1, *p* = 0.005 compared to controls; Fig. 3C). Similarly, in PMA-treated K562 cells, *NR4A3* upregulation could be blocked by siRNA pre-treatment. *NR4A3* knockdown K562 cells also exhibited minimal *PDGFA* upregulation after PMA treatment (*p* = 0.014, Fig. 3E). These findings demonstrated that *NR4A3* knockdown in myeloid leukemia cell lines could attenuate chemically induced differentiation. Thus, downregulation of *NR4A3* expression may contribute to myeloid leukemogenesis by impairing cellular differentiation.

### Association of NR4A3 expression and cell differentiation in clinical samples

**Fig. 4:**
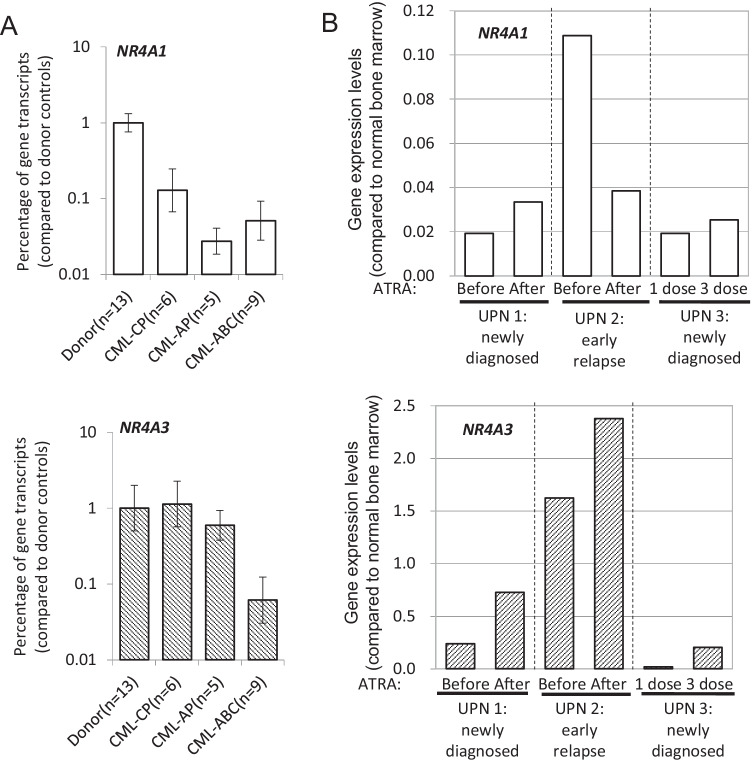
Association between *NR4A3* expression and cell differentiation in clinical samples. **A** *NR4A1* and *NR4A3* gene expression were measured by qPCR in bone marrow samples of patients with chronic myeloid leukemia (CML) in chronic phase (CP), accelerated phase (AP), and acute blast change (ABC). **B** Relative *NR4A1* and *NR4A3* expression levels of peripheral blood samples for three APL patients before and after ATRA administration. The data in both (**A**) and (**B**) were normalized to the expression level of the same gene in normal marrow cells from healthy bone marrow transplantation donors. Abbreviation: UPN, unique patient number

To corroborate what we had observed in cell models, we assayed *NR4A3* expression in archived bone marrow samples from patients with various stages of CML and peripheral blood samples from patients with APL. Compared with healthy marrow donors, chronic phase CML (CML-CP) patients had lower expression of *NR4A1* but not *NR4A3*. This result is compatible with the idea that downregulation of *NR4A1* confers a proliferation advantage (class I aberration), resulting in a myeloproliferative presentation. With disease progression to accelerated phase (CML-AP) and then to acute blastic change (CML-ABC) stages, the *NR4A3* expression levels were gradually decreased, consistent with the expectation for a class II aberration in leukemic cells with progressive differentiation impairment (Fig. 4A).

Since the primary treatment for APL is differentiation therapy, we tested *NR4A1* and *NR4A3* expression levels in three patients before and after ATRA-based treatment. Remarkably, in all three patients, *NR4A3* expression levels were relatively higher after the ATRA leukemia cell differentiation treatments (Fig. 4B), whereas the expression levels of *NR4A1* were not consistently or overtly altered. These data were again in line with our central conclusion that *NR4A3* is tightly linked to cellular differentiation, and its downregulation comprises a class II aberration in myeloid leukemogenesis.

## Discussion

Our results in this study suggest that downregulation of *NR4A3* expression impairs myeloid cell differentiation, an effect that is non-overlapping with that of *NR4A1* downregulation. Combined with the known proliferation-promoting effects of *NR4A1* downregulation, the current findings can be incorporated into the classical two-hit leukemogenic framework to explain why concurrent abrogation of *NR4A1* and *NR4A3* in mice might result in AML-like phenotypes [9, 17].

The mechanism by which *NR4A3* downregulation influences myeloid differentiation remains to be dissected. However, the action is expected to occur early in myeloid differentiation because induction of K562 cell differentiation into different lineages with various compounds yielded similar patterns of *NR4A3* upregulation. Furthermore, these observations suggest that the function of *NR4A3* is probably not associated with a single, specific differentiation program. In line with this notion, our preliminary RNA-seq data from NB4 cells before and after *NR4A3* knockdown showed positive enrichment of both myeloid and erythroid differentiation-related genes (Supplementary Fig. 2). Prior reports showed that *NR4A3* downregulation is a universal phenomenon in every subtype of AML, and in the current study, we show that *NR4A3* downregulation is also associated with the acute transformation of CML. Together, these findings lead us to speculate that downregulation of *NR4A3* is a universal phenomenon and a crucial player during myeloid leukemogenesis.

As yet, the molecular actions of NR4A3 protein are not well described, but there is some evidence that the protein may be maintained at low levels in the cell. In fact, we performed preliminary experiments overexpressing GFP-tagged NR4A3 protein in K562 and NB4 cells, but we ultimately failed to detect fluorescent signals in the cells. We also expressed the protein in 293 T cells but could only detect the signal in cells co-treated with the proteasome inhibitor bortezomib, suggesting that the overexpressed protein might be rapidly degraded (data not shown). In line with this idea, the initial report of *NR4A3* downregulation in myeloid leukemogenesis only included measurements of gene expression levels rather than protein levels [9]. This is also true of studies probing *NR4A3* expression in eosinophils of patients with atopic dermatitis [16, 27]. Based on the paucity of reports detecting endogenous protein with conventional immunohistochemistry or immunoblotting methods, it is possible that high turnover of NR4A3 might be an important regulatory mechanism of protein activity.

Because no mutations in *NR4A3* have been reported, the gene is probably not downregulated by loss-of-function mutations. However, it has been suggested that DNA methylation might contribute to *NR4A3* downregulation [28]. In addition to this potential mechanism, *NR4A3* was found to be a downstream target of *RUNX1* [29], a gene in which class II aberrations frequently occur in AML. As such, *NR4A3* downregulation may link the *RUNX1* class II mutation with its cellular effect of impaired differentiation. In the present study, we observed that *NR4A3* expression increases in APL patients after administration of ATRA-based differentiation therapy. Together, the findings imply that *NR4A3* expression might be regulated by multiple essential AML-associated genes, and it could serve as a common downstream effector of several different class II genetic aberrations.

The potential role of *NR4A3* downregulation in APL is especially intriguing. In the initial paper showing that coincident downregulation of *NR4A3* and *NR4A1* is sufficient to cause AML [9], several APL cases were included in the group of samples used for clinical validations. Consistent with the other samples used for clinical validation, the APL samples had lower *NR4A3* expression levels than controls. Our experiments with NB4 cells (APL cell line with t(15;17)) and patient samples also showed that ATRA-induced differentiation consistently causes *NR4A3* upregulation. Furthermore, suppressing *NR4A3* upregulation in NB4 cells attenuated the ATRA-induced differentiation phenotype. These findings are compatible with the notion that downregulation of *NR4A3* is associated with impaired differentiation in APL. One possible mechanism that may link *NR4A3* downregulation with APL involves the fusion oncoprotein PML/RARα, which is the product of t(15;17). PML/RARα is known to enhance STAT3 expression [30, 31], and STAT3 has been reported to epigenetically silence *NR4A3* expression in gastric cancer [32]. Thus, PML/RARα might directly cause *NR4A3* downregulation and contribute to the development of APL. Further studies will be needed to assess this possibility.

While our data from ATRA-treated APL patients are limited to only three subjects, we saw that ATRA treatment predictably and consistently led to upregulation of *NR4A3* expression. This result suggests that differentiation treatment may upregulate *NR4A3*, and it warrants future studies to examine the effect in large patient populations. Furthermore, this and our other findings suggest the possibility that *NR4A3*-upregulating therapies may be applicable to myeloid leukemias. As such, restoration of *NR4A3* might be achieved using any of several identified compounds, such as histone deacetylase inhibitors, n-butylenephtalide, or palmitate [33–39]. Moreover, NR4A3 agonists have also been identified, including 6-mercaptopurine or prostaglandin A2 [40–43]. Thus, it may be possible to target either the gene expression level or activity of NR4A3 as a strategy for AML treatment.

## Conclusions

Overall, our study provides new cellular and clinical data supporting the idea that downregulation of *NR4A3* is functionally associated with impaired differentiation in myeloid leukemias. In light of the fact that *NR4A1* downregulation provides a cell proliferation advantage, this role for *NR4A3* is well-aligned with the two-hit hypothesis of myeloid leukemogenesis. Such an explanation can reasonably explain why AML would develop in mice with concurrent abrogation of *NR4A1* and *NR4A3*. In addition, the assignment of *NR4A1* as a class I aberration and *NR4A3* downregulation as a class II aberration suggests that the double-knockout animal model might faithfully recapitulate key aspects of the myeloid leukemogenesis process in humans, and it could be an excellent explorative model for further AML research. Moreover, our data suggest that targeting *NR4A3* upregulation might yield novel differentiation therapies, and such treatments could have potential to be clinically beneficial for myeloid leukemias.

## Supplementary information

Below is the link to the electronic supplementary material.

Supplementary file1 (PDF 64 KB)

Supplementary file2 (PDF 53 KB)

## Data Availability

The datasets used and/or analyzed during the current study are available from the corresponding author on reasonable request.
