# Supplementary material for: Functional association of NR4A3 downregulation with impaired differentiation in myeloid leukemogenesis
Source: Ann Hematol. 2022 Aug 30;101(10):2209–18. doi: 10.1007/s00277-022-04961-1 (PMC9463347; doi:10.1007/s00277-022-04961-1)
Supplement: Supplementary file 2 — Supplementary file2 (PDF 53 KB) [file 277_2022_4961_MOESM2_ESM.pdf]

MYELOID CELL DIFFERENTIATION

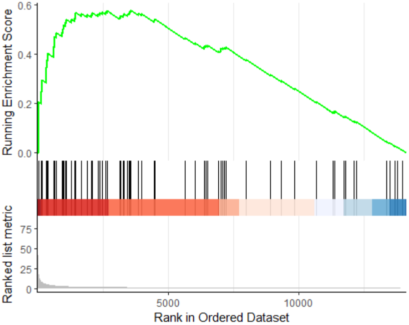

enrichmentScore	NES	pvalue
0.5766919	1.796657	0.002522233

ERYTHROCYTE DIFFERENTIATION

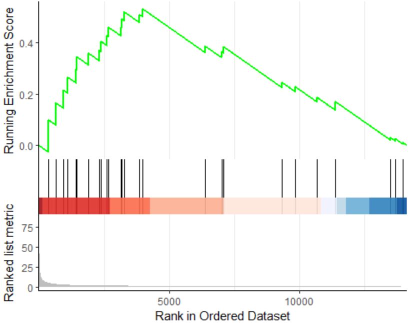

enrichmentScore	NES	pvalue
0.5306659	1.4996	0.09897959
