# Supplementary material for: Functional association of NR4A3 downregulation with impaired differentiation in myeloid leukemogenesis
Source: Ann Hematol. 2022 Aug 30;101(10):2209–18. doi: 10.1007/s00277-022-04961-1 (PMC9463347; doi:10.1007/s00277-022-04961-1)
Supplement: Supplementary file 1 — Supplementary file1 (PDF 64 KB) [file 277_2022_4961_MOESM1_ESM.pdf]

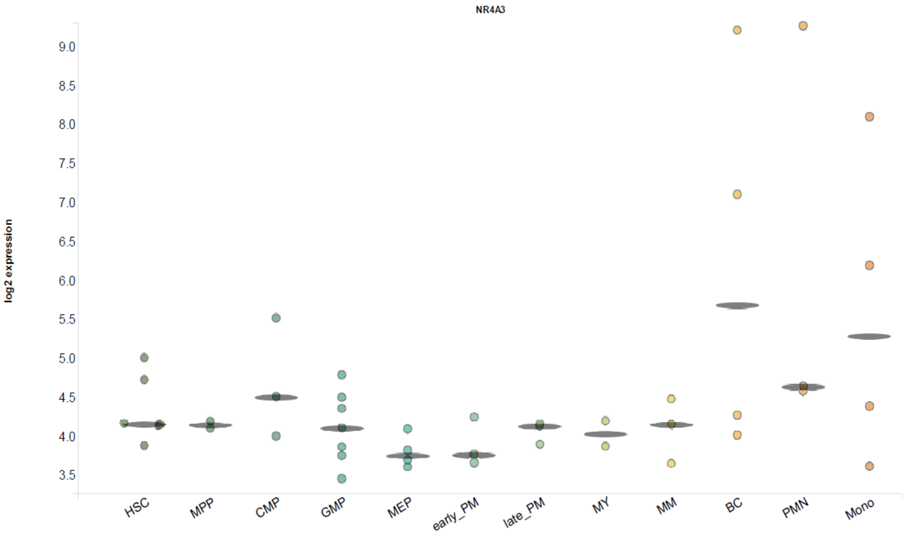


| | |
|---|---|
| HSC | Hematopoietic stem cell |
| MPP | Multipotential progenitors |
| CMP | Common myeloid progenitor cell |
| GMP | Granulocyte monocyte progenitors |
| MEP | Megakaryocyte-erythroid progenitor cell |
| early_PM | Early Promyelocyte |
| late_PM | Late Promyelocyte |
| MY | Myelocyte |
| MM | Metamyelocytes |
| BC | Band cell |
| PMN | Polymorphonuclear cells |
| Mono | Monocytes |
